# MiR-29b-3p promotes particulate matter-induced inflammatory responses by regulating the C1QTNF6/AMPK pathway

**DOI:** 10.18632/aging.102672

**Published:** 2020-01-18

**Authors:** Jian Wang, Mengchan Zhu, Ling Ye, Cuicui Chen, Jun She, Yuanlin Song

**Affiliations:** 1 Department of Pulmonary Medicine, Zhongshan Hospital, Fudan University, Shanghai 200030, China

**Keywords:** particulate matter, miR-29b-3p, complement C1q tumor necrosis factor-related protein 6, AMPK pathway, inflammatory responses

## Abstract

Inflammatory responses are considered to be the critical mechanism underlying particulate matter (PM)-induced development and exacerbation of chronic respiratory diseases. MiR-29b-3p has been found to participate in various biological processes, but its role in PM-induced inflammatory responses was previously unknown. Here, we constructed a miRNA PCR array to find that miR-29b-3p was the most highly expressed in human bronchial epithelial cells (HBECs) exposed to PM. MiR-29b-3p promoted PM-induced pro-inflammatory cytokines (IL-1β, IL-6, and IL-8) expression via inhibiting the AMPK signaling pathway in HBECs. RNA sequencing and luciferase reporter assay identified that miR-29b-3p targeted complement C1q tumor necrosis factor-related protein 6 (C1QTNF6), a protein that protected from PM-induced inflammatory responses via activating the AMPK signaling pathway. *In vivo*, miR-29b-3p antagomirs delivered via the tail vein prior to PM exposure significantly counteracted PM-induced miR-29b-3p upregulation and C1QTNF6 downregulation in lung tissues. Furthermore, miR-29b-3p inhibition alleviated inflammatory cells infiltration and pro-inflammatory cytokines secretion in the lung of PM-exposed mice. These findings firstly revealed that miR-29b-3p acted as a novel modulator of PM-induced inflammatory responses by targeting the C1QTNF6/AMPK signaling pathway, which contributes to a better understanding of the biological mechanisms underlying adverse PM-induced respiratory health effects.

## INTRODUCTION

Ambient particulate matter (PM) is the key component of air pollution, which is a great threat to global health, especially in developing countries. The Global Burden of Diseases, Injuries, and Risk Factors Study 2017 (GBD 2017) showed that PM exposure caused 4.58 million deaths and 143 million disability-adjusted life-years (DALYs) across the world in 2017 [1]. Recently, increasing epidemiologic studies have demonstrated that PM exposure is closely associated with morbidity and mortality due to chronic respiratory diseases, such as chronic obstructive pulmonary diseases (COPD) and asthma [2]. Inflammatory responses are considered to be the main biological mechanism underlying the adverse PM-induced respiratory health effects. A panel study reported that fine PM exposure elevated the levels of several pro-inflammatory cytokines in the blood of healthy young adults with no history of smoking [3]. Moreover, numerous studies have shown that PM exposure promotes the expression of pro-inflammatory cytokines in human bronchial epithelial cells (HBECs), alveolar epithelial cells, and macrophages [4, 5]. The infiltration of inflammatory cells around PM deposits and the secretion of pro-inflammatory cytokines in lung tissues of PM-exposed mice have also been reported [5]. Oxidative stress and several signaling pathways have been shown to regulate PM-induced inflammatory responses [6]. In our previous studies, proteins (amphiregulin and osteopontin) were found to participate in regulating PM-induced production of pro-inflammatory cytokines (IL-1α and IL-1β) in HBECs [7, 8]. However, the previous research findings could not completely elucidate the regulatory mechanism underlying PM-induced inflammatory responses. Moreover, the potential treatment targets to prevent adverse PM-induced respiratory effects were also lacking.

MicroRNAs (miRNAs) are small non-coding RNAs of 20–24 nucleotides in length that mediate gene silencing by binding to the 3′ untranslated region (UTR) of mRNAs [9]. Accumulating evidence showed that PM exposure changed several inflammation-associated miRNAs expression in human peripheral blood samples [10]. Chen et al. showed that fine PM exposure reduced the expression of several miRNAs (miR-21-5p, miR-187-3p, miR-146a-5p, miR-1-3p, and miR-199a-5p), which contributed to increased inflammation, coagulation, and vasoconstriction in the peripheral blood of healthy young volunteers [11]. Bollati et al. found that PM exposure increased miR-222 and miR-21 expression in peripheral blood leukocytes of workers and these two miRNAs were demonstrated to be associated with inflammation and oxidative stress [12]. Motta et al. used high-throughput array analysis to find that four inflammation-related miRNAs (miR-421, miR-29a, miR-146a, and let-7g) were significantly upregulated in humans with metal-rich PM exposure [13]. Additionally, several studies have reported changes in miRNA expression profiles in various lung cells exposed to PM [14, 15]. The microarray analysis showed that seasonal urban PM increased the expression of several miRNAs (miR-146a, miR-1246 and miR-29c), which were associated with inflammation, oxidative stress and epigenetic modification in BEAS2-B cells [15]. However, a limited number of studies have explored the functional effects of miRNAs on PM-induced inflammatory responses. Song et al. showed that fine PM downregulated the expression of miR-331 and thereby promoted inflammatory responses via activation of NF-κB pathway [16]. Actually, the regulatory roles of the most miRNAs in PM-induced inflammatory responses were previously largely unknown.

The present study aimed to investigate the effects of miRNAs on PM-induced inflammatory responses. We screened for 92 miRNAs related to inflammation in HBECs exposed to PM 1649b and found that miR-29b-3p is the most highly upregulated miRNA with high abundance. Our data demonstrated that miR-29b-3p promoted PM-induced inflammatory responses by targeting complement C1q tumor necrosis factor-related protein 6 (C1QTNF6) and inhibiting the activation of the protective AMPK pathway. These results indicated that miR-29b-3p was an important interventional target in PM-induced inflammatory responses.

## RESULTS

### MiR-29b-3p expression was upregulated upon PM exposure

**Figure 1:**
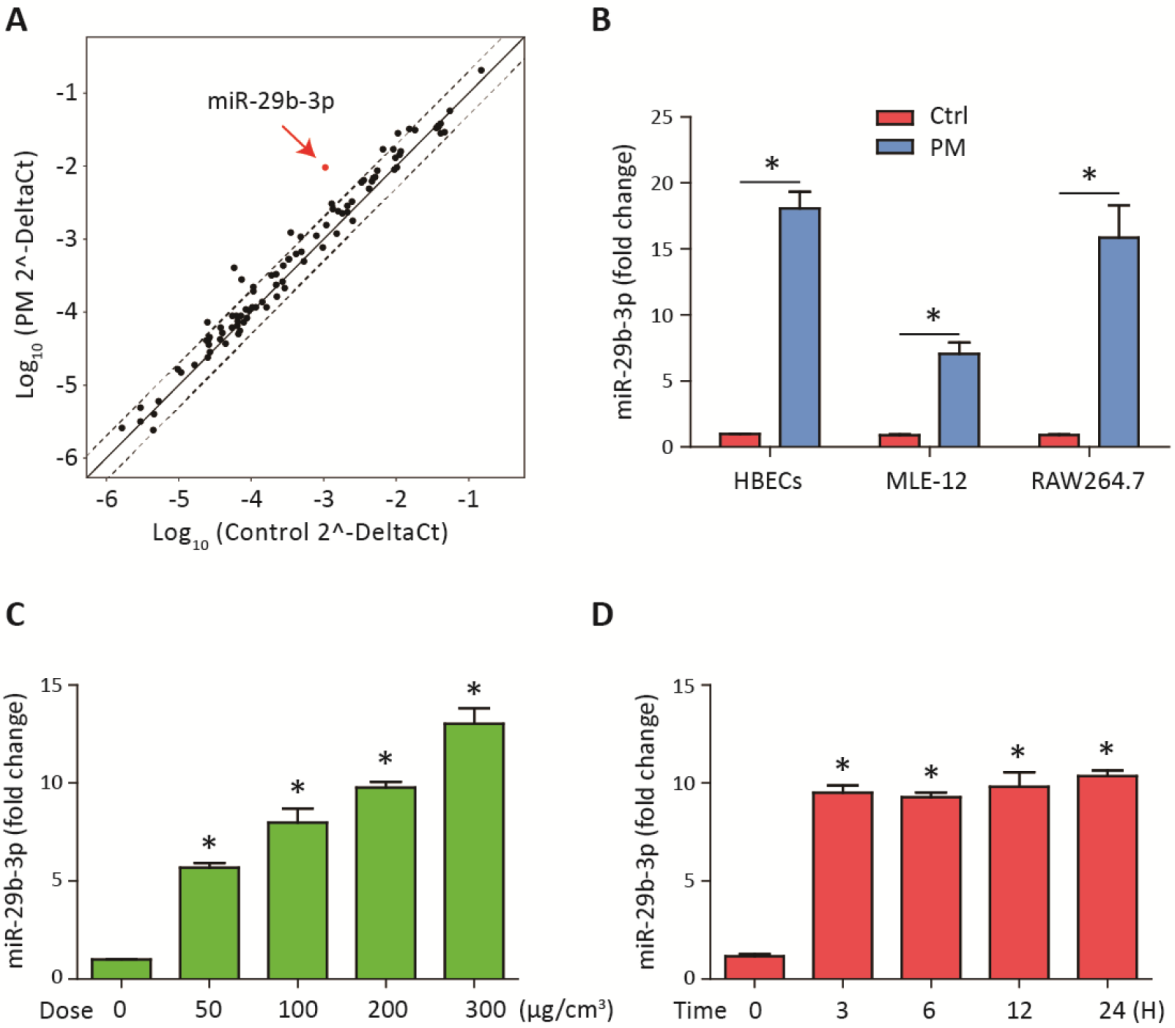
**MiR-29b-3p was upregulated upon PM exposure.** (**A**) The expression of miRNAs was measured using a inflammation-related miRNAs PCR array in HBECs with or without 300 μg/cm^3^ PM for 24 h. The points outside the dashed lines indicated miRNAs with fold change > 2 among the subsets (n= 2 biological replicates). (**B**) Real-time PCR analysis of miR-29b-3p expression in HBECs, MLE-12, and RAW264.7 cells with or without 300 μg/cm^3^ PM for 24 h. (**C**) HBECs were stimulated with different doses of PM (50, 100, 200, and 300 μg/cm^3^) for 24 h and the expression of miR-29b-3p was detected using real-time PCR. (**D**) HBECs were treated with 300 μg/cm^3^ PM for different durations (3, 6, 12, and 24 h) and the expression of miR-29b-3p was detected using real-time PCR. Values represent mean ± SEM; *, P<0.05, compared with the control group; n=3. HBECs, human bronchial epithelial cells; PM, particulate matter.

To investigate the differential expression of miRNAs after PM-induced inflammation, the expression profiles of miRNAs in HBECs stimulated with 300 μg/cm^3^ PM for 24 h were analyzed using a inflammation-related miRNAs PCR array. We found that miR-29b-3p was the most highly upregulated in the PM-exposed HBECs (Figure 1A). To further validate the results from miRNAs PCR array, the expression of miR-29b-3p in HBECs, MLE-12 and RAW264.7 was determined. PM exposure significantly increased the expression of miR-29b-3p in these three cells (Figure 1B). When HBECs were stimulated with different doses of PM (50, 100, and 300 μg/cm^3^) over 24 h, the expression of miR-29b-3p was significantly upregulated in a dose-dependent manner (Figure 1C). In addition, when HBECs were stimulated with 300 μg/cm^3^ PM for different durations (3, 6, 12, and 24 h), miR-29b-3p expression was significantly increased in a time-dependent manner (Figure 1D). These findings suggested that miR-29b-3p was involved in the injury process upon PM exposure.

### MiR-29b-3p promoted PM-induced inflammatory responses via inhibiting the activation of AMPK pathway

**Figure 2:**
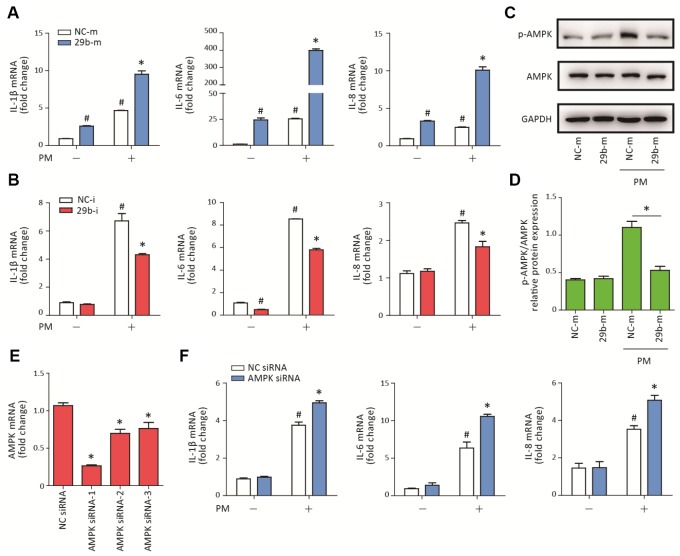
**MiR-29b-3p promoted PM-induced inflammatory responses via repressing AMPK pathway activation.** (**A**) HBECs were transfected with miR-29b-3p mimic (29b-m) or negative control mimic (NC-m), and then treated with or without 300 μg/cm^3^ PM for 24 h. Real-time PCR analysis of IL-1β, IL-6, and IL-8 expression in HBECs transfected with 29b-m or NC-m prior to PM exposure. Values represent mean ± SEM; *, P<0.05, compared with the NC-m + PM group; #, P<0.05, compared with the NC-m group; n=3. (**B**) HBECs were transfected with miR-29b-3p inhibitor (29b-i), or negative control inhibitor (NC-i), and then treated with or without 300 μg/cm^3^ PM for 24 h. Real-time PCR analysis of IL-1β, IL-6, and IL-8 expression in HBECs transfected with 29b-i or NC-i prior to PM exposure. Values represent mean ± SEM; *, P<0.05, compared with the NC-i + PM group; #, P<0.05, compared with the NC-i group; n=3. (**C**) Western blot analysis of AMPK signaling pathway activation in HBECs transfected with 29b-m or NC-m prior to PM exposure. The optical densities of protein bands were shown in (**D**). Values represent mean ± SEM; *, P<0.05, compared with the NC-m + PM group; n=3. (**E**) The AMPK siRNAs were transfected into HBECs 24h prior to PM exposure, respectively and the optimum AMPK siRNA was selected using real-time PCR. Values represent mean ± SEM; *, P<0.05, compared with the NC siRNA group; n=3. (**F**) Real-time PCR analysis of IL-1β, IL-6, and IL-8 expression in HBECs transfected with AMPK siRNA or negative control siRNA prior to PM exposure. Values represent mean ± SEM; *, P<0.05, compared with the NC siRNA + PM group; #, P<0.05, compared with the NC siRNA group; n=3. HBECs, human bronchial epithelial cells; PM, particulate matter.

To determine the potential role of miR-29b-3p in PM-induced inflammatory responses, miR-29b-3p mimic (29b-m) or miR-29b-3p inhibitor (29b-i) were used to upregulate or downregulate, respectively, the endogenous expression of miR-29b-3p in HBECs prior to PM exposure. After the HBECs were transfected with 29b-m or negative control mimic (NC-m), 29b-m significantly increased the expression of pro-inflammatory cytokines (IL-1β, IL-6, and IL-8) compared to NC-m. Furthermore, 29b-m also significantly potentiated PM-induced IL-1β, IL-6, and IL-8 expression compared to NC-m (Figure 2A). In contrast, after the HBECs were transfected with 29b-i or negative control inhibitor (NC-i), 29b-i could decrease the expression of IL-6, but have no inhibitory effect on the expression of IL-1β and IL-8, compared to NC-i. However, 29b-i significantly inhibited the PM-induced IL-1β, IL-6, and IL-8 expression, compared to NC-i (Figure 2B). These results indicated a pro-inflammatory role of miR-29b-3p in PM-induced inflammatory responses.

The AMPK signaling pathway plays an important role in balancing cellular energy metabolism and has been found to inhibit inflammatory responses in various model systems [17]. To investigate the regulatory effect of miR-29b-3p on the AMPK signaling pathway, the phosphorylation of AMPK was detected in HBECs transfected with 29b-m or NC-m prior to PM exposure. Western blot results showed that 29b-m significantly inhibited the activation of the AMPK signaling pathway compared to NC-m in HBECs upon PM exposure (Figure 2C–2D). To further confirm the role of the AMPK signaling pathway in PM-induced inflammation, AMPK siRNA was used to inhibit the expression of AMPK in HBECs prior to PM exposure. The optimum AMPK siRNA was selected according to the inhibition efficiency of three custom-designed siRNAs using RT-PCR (Figure 2E). Inhibition of the AMPK pathway significantly promoted PM-induced IL-1β, IL-6, and IL-8 expression in HBECs (Figure 2F). These results suggested that miR-29b-3p inhibited the protective AMPK pathway and thereby promoted PM-induced inflammatory responses.

### C1QTNF6 is the downstream target of miR-29b-3p

**Figure 3:**
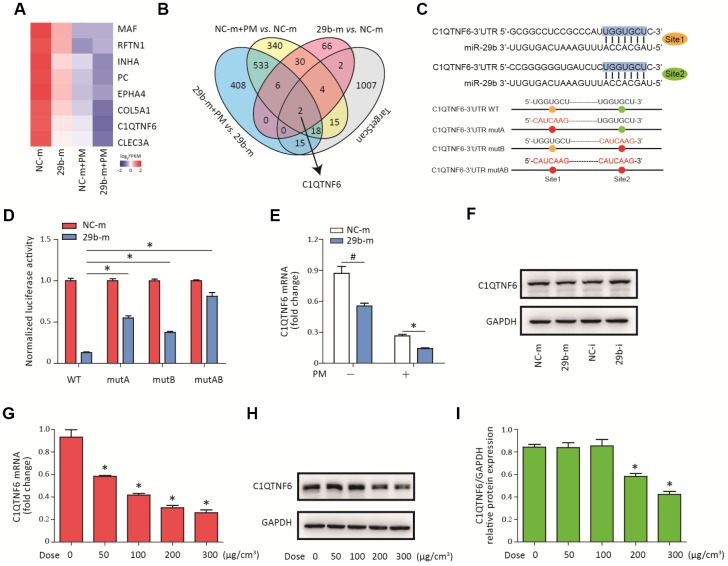
**C1QTNF6 is the target gene of miR-29b-3p.** (**A**) HBECs were transfected with miR-29b-3p mimic (29b-m) or negative control mimic (NC-m), respectively, and then treated with or without 300 μg/cm^3^ PM for 24 h. RNA sequencing identified the differentially-expressed genes in HBECs in the four groups (NC-m, 29b-m, NC-m + PM, and 29b-m + PM). The heatmap identified eight differentially downregulated genes common to the NC-m vs. 29b-m groups, NC-m + PM vs. 29b-m + PM groups, and NC-m vs. NC-m + PM groups. (**B**) Venn diagram showed the common differentially-downregulated genes in RNA sequencing and TargetScan analysis. (**C**) The binding sites between miR-29b-3p and the 3'UTR of C1QTNF6 were predicted by TargetScan. The aligned sequences of the 3'UTR of C1QTNF6 complementary to the seed sequence of miR-29b-3p and mutant sequences were shown. (**D**) HBECs were transfected with C1QTNF6-3'-UTR-WT, C1QTNF6-3'-UTR-mutA, C1QTNF6-3'-UTR-mutB or C1QTNF6-3'-UTR-mutAB plasmids combined with 29b-m or NC-m, respectively. The normalized luciferase activities were determined by luciferase reporter assay. Values represent mean ± SEM; *, P<0.05, compared with the WT plasmid + 29b-m group; n=6. (**E**) Real-time PCR analysis of C1QTNF6 expression in HBECs transfected with 29b-m or NC-m prior to PM exposure. Values represent mean ± SEM; *, P<0.05, compared with the NC-m + PM group; #, P<0.05, compared with the NC-m group; n=3. (**F**) Western blot analysis of C1QTNF6 expression in HBECs transfected with 29b-m, NC-m, miR-29b-3p inhibitor (29b-i), or negative control inhibitor (NC-i), respectively. (**G**) HBECs were stimulated with different doses of PM (50, 100, 200, and 300 μg/cm^3^) for 24 h and the mRNA expression of C1QTNF6 was detected using real-time PCR. (**H**) The protein expression of C1QTNF6 was detected using western blot analysis. The optical densities of protein bands were shown in (**I**). Values represent mean ± SEM; *, P<0.05, compared with the control group; n=3. HBECs, human bronchial epithelial cells; PM, particulate matter.

To determine the molecular target of miR-29b-3p, RNA sequencing was used to detect the gene expression profiles of HBECs in four groups (NC-m, 29b-m, NC-m + PM, and 29b-m + PM). Eight differentially downregulated genes common to the NC-m *vs.* 29b-m groups, NC-m + PM *vs.* 29b-m + PM groups, and NC-m *vs.* NC-m + PM groups were identified (Supplementary Figure 1). The heatmap in Figure 3A showed the expression level of these eight genes in the four groups. Next, two genes (C1QTNF6 and COL5A1) were predicted to be the regulatory targets of miR-29b-3p based on the TargetScan database (Figure 3B). According to previous research, C1QTNF6 is an important member of C1QTNF family and has been found to exert anti-inflammatory effects in several disease models [18]. Furthermore, two conserved sequences complementary to the seed sequence of miR-29b-3p in the 3'UTR of C1QTNF6 were predicted by the TargetScan database (Figure 3C). To further confirm the interaction between miR-29b-3p and C1QTNF6, three different mutant plasmids (C1QTNF6-3'-UTR-mutA, C1QTNF6-3'-UTR-mutB, and C1QTNF6-3'-UTR-mutAB) or a WT plasmid (C1QTNF6-3'-UTR-WT) were transferred to cells combined with either 29b-m or NC-m. The dual luciferase reporter gene assay showed that miR-29b-3p overexpression significantly inhibited the reporter activity in the WT C1QTNF6 3'UTR group and this effect was abolished when using the three different C1QTNF6 3'UTR mutants, especially the C1QTNF6-3'-UTR-mutAB (Figure 3D). Moreover, miR-29b-3p overexpression inhibited the expression of C1QTNF6 mRNA and protein, while miR-29b-3p inhibition promoted the expression of C1QTNF6 protein in HBECs (Figure 3E–3F and Supplementary Figure 2). Additionally, RT-PCR showed that PM exposure significantly inhibited C1QTNF6 mRNA expression in a dose-dependent manner in HBECs (Figure 3G). The inhibitory effect of PM on the protein expression of C1QTNF6 was also confirmed (Figure 3H–3I). Thus, these findings suggested that C1QTNF6 was the potential target of miR-29b-3p.

### C1QTNF6 overexpression attenuated PM-induced inflammatory responses

**Figure 4:**
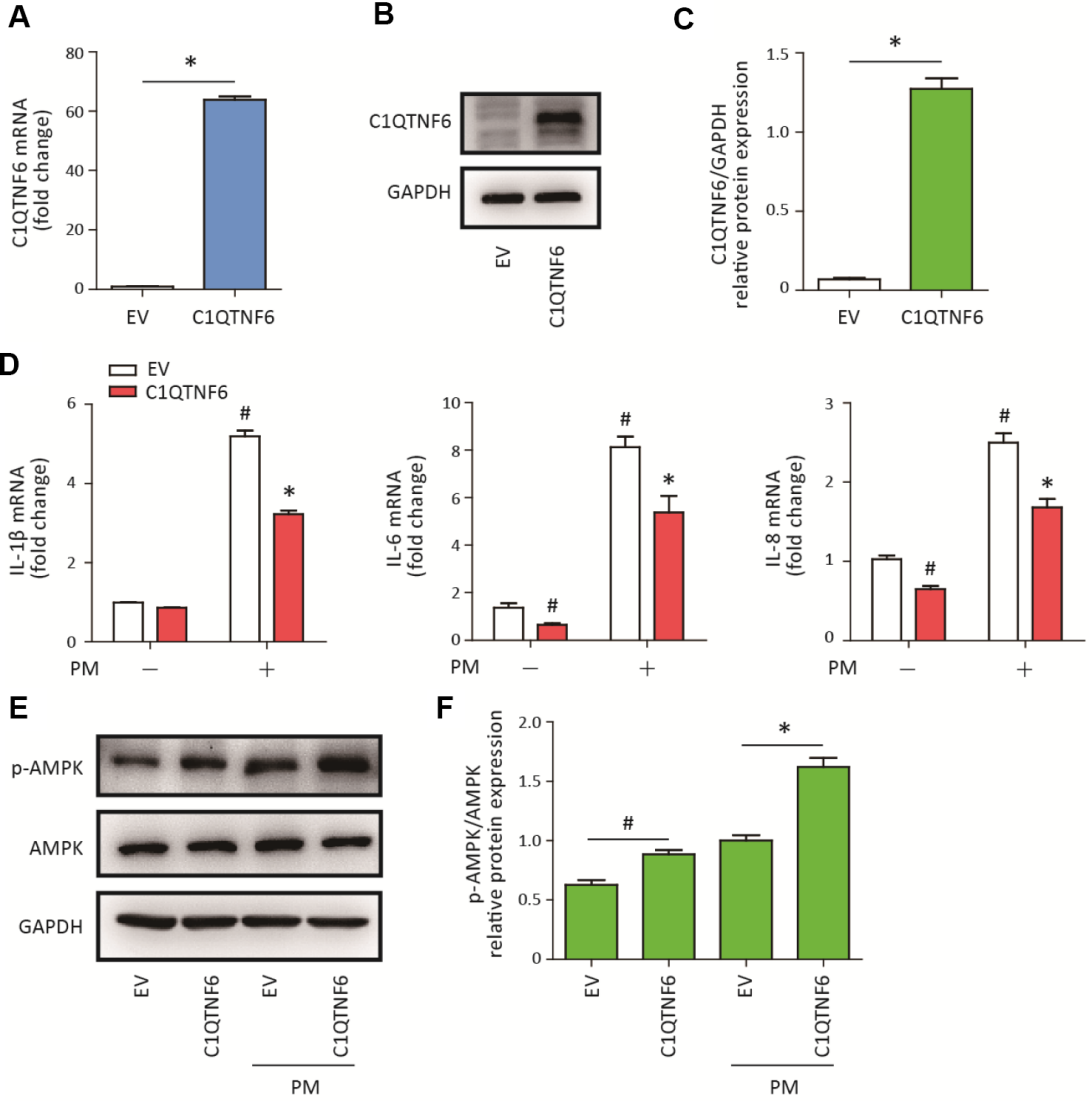
**C1QTNF6 overexpression attenuated PM-induced inflammatory responses.** HBECs were transfected with C1QTNF6-overexpressing virus or negative control virus (EV). The C1QTNF6 overexpression efficiency was confirmed using real-time PCR (**A**) and western blot analysis (**B**). The optical densities of protein bands were shown in (**C**). Values represent mean ± SEM; *, P<0.05, compared with the EV group; n=3. (**D**) Real-time PCR analysis of IL-1β, IL-6, and IL-8 expression in C1QTNF6-overexpressing or negative control HBECs treated with or without 300 μg/cm^3^ PM for 24 h. (**E**) Western blot analysis of AMPK signaling pathway activation in C1QTNF6-overexpressed or negative control HBECs treated with or without 300 μg/cm^3^ PM for 24 h. The optical densities of protein bands were shown in (**F**). Values represent mean ± SEM; *, P<0.05, compared with the EV + PM group; #, P<0.05, compared with the EV group; n=3. EV, empty vector; HBECs, human bronchial epithelial cells; PM, particulate matter.

To determine the role of C1QTNF6 in PM-induced inflammatory responses in HBECs, C1QTNF6-overexpressing cells were constructed. RT-PCR and western blot analysis showed that C1QTNF6 was significantly upregulated at the mRNA and protein levels in the constructed HBECs (Figure 4A–4C). Next, C1QTNF6 overexpression decreased the expression of IL-6 and IL-8, but had no inhibitory effect on the expression of IL-1β, compared to the negative control cells. When HBECs were stimulated with PM, C1QTNF6 overexpression significantly inhibited PM-induced IL-1β, IL-6, and IL-8 expression in HBECs (Figure 4D). Furthermore, western blot results showed that C1QTNF6 overexpression in HBECs significantly promoted the activation of the AMPK signaling pathway compared to negative control cells exposed to PM (Figure 4E–4F). These results suggested that C1QTNF6 played a protective role in the PM-induced inflammatory responses.

### C1QTNF6 silencing aggravated PM-induced inflammatory responses

**Figure 5:**
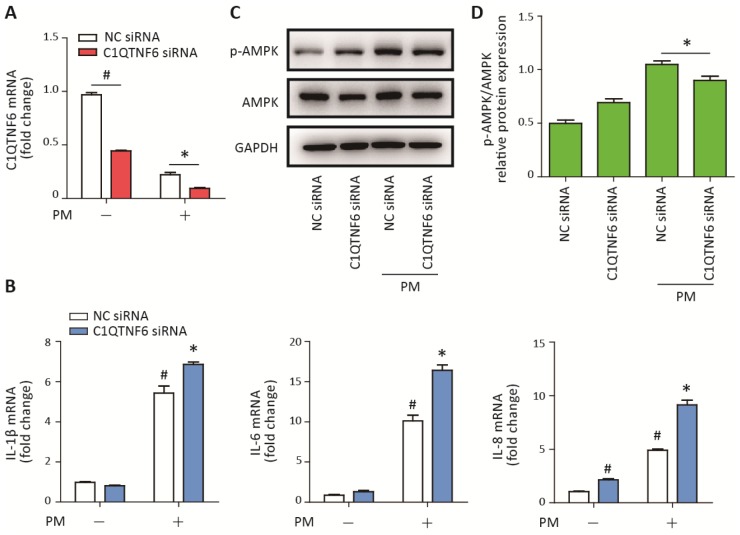
**C1QTNF6 silence enhanced the PM-induced inflammatory responses.** HBECs were transfected with C1QTNF6 siRNA or negative control siRNA, respectively and then treated with or without 300 μg/cm^3^ PM for 24 h. (**A**) Real-time PCR analysis of C1QTNF6 expression in HBECs transfected with C1QTNF6 siRNA or negative control siRNA prior to PM exposure. (**B**) Real-time PCR analysis of IL-1β, IL-6, and IL-8 expression in HBECs transfected with C1QTNF6 siRNA or negative control siRNA prior to PM exposure. (**C**) Western blot analysis of the AMPK signaling pathway activation in HBECs transfected with C1QTNF6 siRNA or negative control siRNA prior to PM exposure. The optical densities of protein bands were shown in (**D**). Values present as mean ± SEM; *, P<0.05, compared with the NC siRNA + PM group; #, P<0.05, compared with the NC siRNA group; n=3. HBECs, human bronchial epithelial cells; PM, particulate matter.

To further confirm the anti-inflammatory effect of C1QTNF6, C1QTNF6 siRNA was used to reduce the expression of C1QTNF6 in HBECs prior to PM exposure. The optimum C1QTNF6 siRNA was identified according to the inhibition efficiencies of three custom-designed siRNAs using RT-PCR (Supplementary Figure 3). Moreover, RT-PCR showed that C1QTNF6 siRNA significantly inhibited the expression of C1QTNF6 in HBECs with or without PM exposure (Figure 5A). C1QTNF6 inhibition increased the expression of IL-8, but had no effect on the expression of IL-1β and IL-6, compared to the negative control (Figure 5B). When cells were stimulated with PM, C1QTNF6 inhibition significantly potentiated PM-induced IL-1β, IL-6, and IL-8 expression in HBECs (Figure 5B). Additionally, western blot results showed that the AMPK signaling pathway was inhibited by C1QTNF6 silencing in HBECs exposed to PM, compared to the negative control (Figure 5C, 5D). These data further confirmed that C1QTNF6 was an anti-inflammatory molecule in PM-induced inflammatory responses.

### MiR-29b-3p inhibition alleviated the PM-induced acute lung inflammatory responses *in vivo*

**Figure 6:**
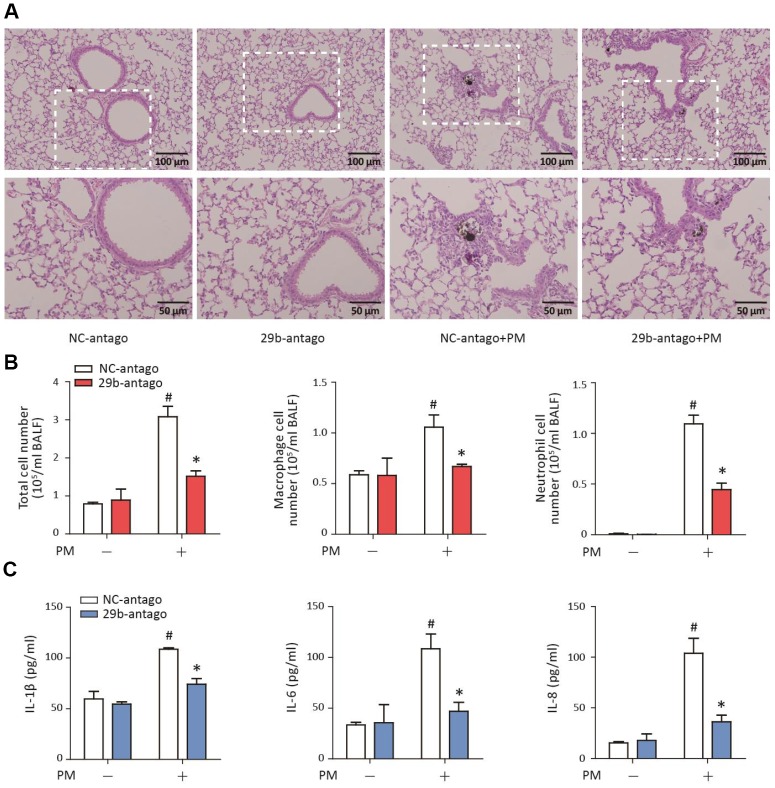
**MiR-29b-3p inhibition attenuated the PM-induced acute lung inflammatory responses *in vivo*.** The acute PM-exposed mouse model was constructed and miR-29b-3p antagomirs (29b-antago) or negative control antagomirs (NC-antago) were delivered via the tail vein of mice 24 h prior to the first PM exposure. (**A**) The histopathologic analysis of acute inflammatory responses in lung tissue of mice using H&E staining. (**B**) The numbers of total cells, macrophages and neutrophils in the BALF of mice were counted. (**C**) The concentrations of IL-1β, IL-6, and IL-8 in the BALF of mice were measured by ELISA. Values represent mean ± SEM; *, P<0.05, compared with the NC-antago + PM group; #, P<0.05, compared with the NC-antago group; n=3. BALF, bronchoalveolar lavage fluid; PM, particulate matter.

To determine the role of miR-29b-3p in the PM-induced acute lung inflammatory responses *in vivo*, miR-29b-3p antagomirs (29b-antago) or negative control antagomirs (NC-antago) were injected via the tail vein prior to PM exposure in mice. Histopathologic analyses showed that 29b-antago attenuated acute inflammatory responses and reduced inflammatory cells infiltration around PM deposits in the lung tissues of mice (Figure 6A). In accordance with the histological analysis, the numbers of total cells, macrophages and neutrophils were significantly reduced in the BALF of PM-exposed mice treated with 29b-antago compared to NC-antago (Figure 6B). Additionally, 29b-antago treatment significantly abolished the PM-induced IL-1β, IL-6, and IL-8 secretion in the bronchoalveolar lavage fluid (BALF) of mice (Figure 6C). These results suggested that inhibition of miR-29b-3p protected against the PM-induced acute lung inflammatory responses *in vivo*.

### MiR-29b-3p inhibited the expression of C1QTNF6 *in vivo*

**Figure 7:**
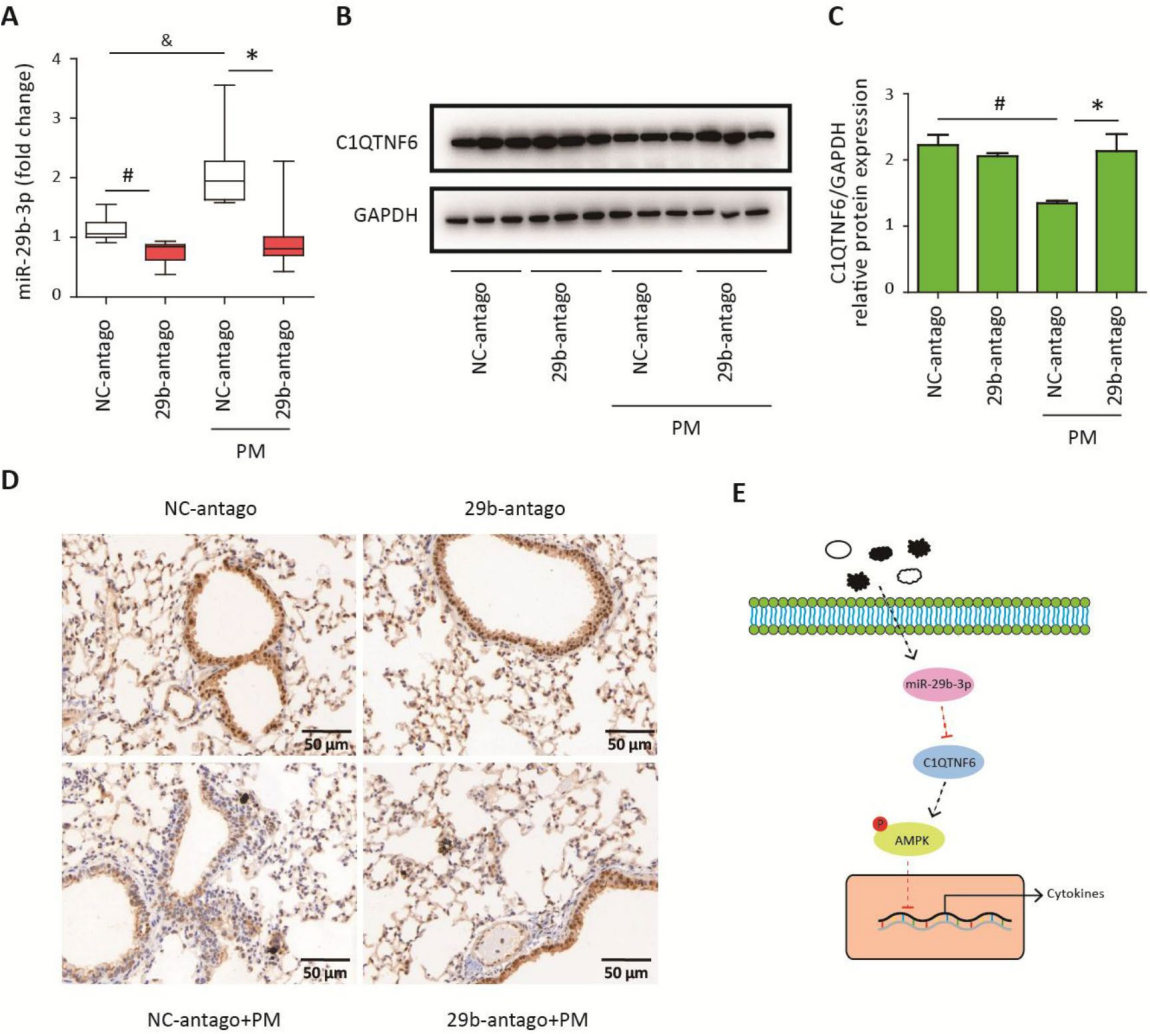
**MiR-29b-3p inhibited the expression of C1QTNF6 *in vivo*.** The acute PM-exposed mouse model was constructed and miR-29b-3p antagomirs (29b-antago) or negative control antagomirs (NC-antago) were delivered through the tail vein of mice 24 h prior to the first PM exposure. (**A**) Real-time PCR analysis of C1QTNF6 expression in mouse models treated with 29b-antago or NC-antago. (**B**) Western blot analysis of C1QTNF6 expression in mouse models treated with 29b-antago or NC-antago. The optical densities of protein bands were shown in (**C**). Values represent mean ± SEM; *, P<0.05, compared with the NC-antago + PM group; #, &, P<0.05, compared with the NC-antago group; n=3-5. (**D**) Immunohistological analysis of C1QTNF6 expression in lung tissue of mouse models treated with 29b-antago or NC-antago. (**E**) Schematic diagram of the critical role of miR-29b-3p in the PM-induced inflammatory responses. miR-29b-3p was upregulated by PM exposure and inhibited the activation of AMPK pathway via targeting C1QTNF6 to promote PM-induced inflammatory responses. PM, particulate matter.

To confirm that 29b-antago exerted an anti-inflammatory effect by inhibiting the expression of C1QTNF6, the lung tissues of mice were collected for further analysis. RT-PCR analysis showed that 29b-antago treatment significantly reduced the expression of miR-29b-3p in the lung tissues of mice. Moreover, PM exposure contributed to the increased expression of miR-29b-3p, which was consistent with the *in vitro* results (Figure 7A). Western blot analysis showed that PM exposure significantly inhibited the protein expression of C1QTNF6 in the lung tissues, and 29b-antago treatment reversed the PM-induced down- regulation of C1QTNF6 (Figure 7B–7C). Similarly, immunohistological analysis confirmed the inhibitory effect of PM exposure on the expression of C1QTNF6, which was abolished by 29b-antago treatment (Figure 7D). Taken together, these results suggested that miR-29b-3p targeted C1QTNF6 expression to promote PM-induced inflammatory responses.

## DISCUSSION

Inflammation is the main biological response to PM exposure and has been considered to be responsible for the PM-induced development and exacerbation of chronic respiratory diseases [7]. This study revealed a critical role of miR-29b-3p in regulating the PM-induced inflammatory responses. Based on both *in vivo* and *in vitro* PM exposure models, we showed that PM exposure promoted miR-29b-3p expression in airway epithelial cells and macrophages, and miR-29b-3p inhibition significantly attenuated PM-induced pro-inflammatory cytokines (IL-1β, IL-6, and IL-8) and inflammatory cell infiltration. Furthermore, miR-29b-3p directly targeted an anti-inflammatory protein, C1QTNF6, to inhibit the activation of the protective AMPK signaling pathway, thus leading to excessive inflammatory responses upon PM exposure (Figure 7E). MiR-29b-3p is an important member of the miR-29 family and has highly conserved structure in humans, rats, and mice [19, 20]. The target genes of miR-29b-3p participate in various biological processes, including cell proliferation, apoptosis, fibrosis and inflammation [21, 22]. Recently, the regulatory roles of miR-29b-3p in inflammatory responses were found to be inconsistent in different disease models. Lu et al. found that miR-29b-3p increased inflammation and oxidative stress by targeting SPPY1 to further promote MAPK signaling pathway activation in atherosclerosis [23]. Zhang et al. also showed that miR-29b-3p was negatively regulated by long non-coding RNA TUG1 and promoted apoptosis and inflammatory responses by activating the NF-κB and JAK/STAT signaling pathways in H9c2 cardiomyoblasts treated with LPS [24]. Similarly, miR-29b enhanced LPS-induced inflammation by activating the NF-κB and JAK/STAT signaling pathways in endothelial cells [25]. However, Dai et al. found that miR-29b-3p suppressed apoptosis and inflammatory responses by inhibiting TRAF3 in intestinal ischemia/reperfusion injury [26]. Eken et al. also showed that miR-29b-3p attenuated irradiation-related vascular inflammatory responses by targeting DPP4 and PTX3 [27]. Furthermore, inhibition of miR-29b-3p increased CDK6 expression and activation of the NF-κB signaling pathway to promote inflammation in IgA nephropathy [28]. In this study, we constructed an miRNA panel to identify that miR-29b-3p was the most highly upregulated in PM-exposed HBECs. Furthermore, we found that miR-29b-3p significantly aggravated PM-induced pro-inflammatory responses via inhibiting C1QTNF6, *in vivo* and *in vitro.* MiR-29b-3p is considered as a potential target for the treatment of PM-induced development and exacerbation of chronic respiratory diseases.

The C1QTNF family consists of 16 members and shares four common domains from the N terminus to the C terminus, comprising a signal peptide, a short variable region, a collagenous domain and a globular domain that is homologous to complement component 1q (C1q) [18]. C1QTNF proteins have been found to form homotrimers or heterotrimers and they participated in regulating metabolism, immunity and inflammation [29]. As a member of the C1QTNF family, C1QTNF6 has been found to play an anti-inflammatory role in most disease models. Murayama et al. found that treatment with recombinant C1QTNF6 attenuated inflammation by preventing excessive complement activation in mouse rheumatoid arthritis models [30]. Chi et al. also found that C1QTNF6 inhibited angiotensin II-induced vascular endothelial inflammation via PPARγ activation [31]. Furthermore, C1QTNF6 promoted the expression of the anti-inflammatory cytokine IL-10 in macrophages and thereby modulated the inflammatory response [32]. However, Lei et al. showed that C1QTNF6 promoted adipose tissue inflammation and insulin resistance in obese and diabetic mouse models [33]. In this study, we showed that C1QTNF6, as the target gene of miR-29b-3p, was significantly down-regulated upon PM exposure *in vivo* and *in vitro.* Overexpression of C1QTNF6 inhibited PM-induced pro-inflammatory cytokine expression, while inhibition of C1QTNF6 enhanced the PM-induced inflammation via modulating AMPK pathway activation in HBECs.

AMPK is an evolutionarily conserved protein kinase and is considered to be a key regulator for balancing cellular energy in eukaryotic cells [34]. Several stimuli activated the AMPK signaling pathway in different cells. Long et al. found that PM_2.5_ increased the activation of the AMPK pathway and autophagy in bronchial epithelial cells [35]. We also found that PM 1649b exposure promoted the activation of the AMPK pathway. Recently, most studies have confirmed that the AMPK pathway plays a protective role in inflammatory responses and, so far, all AMPK activators have been found to exert anti-inflammatory effects in various model systems [17]. For example, Xiang et al. found that activation of the AMPK signaling pathway alleviated IL-1β expression and inhibited the NF-κB activation in inflammatory pain [36]. We also demonstrated that inhibition of the AMPK pathway aggravated PM-induced pro-inflammatory cytokines expression in HBECs and the AMPK pathway was a protective modulator in PM-induced inflammatory responses. The association between C1QTNF6 and AMPK pathway has also been demonstrated in previous studies. Lee et al. showed that C1QTNF6 promoted the activation of the AMPK pathway to modulate fatty acid oxidation in skeletal muscle cells [37]. Similarly, C1QTNF6 exerted anti-fibrotic effect via activation of AMPK pathway in post-infarct cardiac fibrosis [38]. Our results also showed that C1QTNF6 could promote the activation of the AMPK pathway and miR-29b-3p inhibited the activation of AMPK pathway via targeting C1QTNF6 in HBECs upon PM exposure.

In summary, we firstly found that miR-29b-3p was significantly upregulated by PM exposure in airway epithelial cells and alveolar macrophages and miR-29b-3p promoted the expression of pro-inflammatory cytokines (IL-1β, IL-6, and IL-8) both *in vivo* and *in vitro*. Furthermore, miR-29b-3p could directly target C1QTNF6 and thereby inhibited the activation of the anti-inflammatory AMPK signaling pathway during PM-induced inflammatory responses. These results showed, for the first time, that miR-29b-3p served as a vital modulator in PM-induced inflammatory responses, which revealed a new biological mechanism underlying the adverse PM-induced health effects. Moreover, these findings indicated that miR-29b-3p might be a novel therapeutic target for the treatment of adverse PM-induced respiratory effects in the future.

## MATERIALS AND METHODS

### Reagents

Urban PM 1649b was obtained from the National Institute of Standards and Technology (NIST, Gaithersburg, MD, USA). It is a standard reference materials, mainly comprising polycyclic aromatic hydrocarbons (PAHs), polychlorinated biphenyl (PCB), and persistent chlorinated pesticides. Its detailed characteristics are described at www-s.nist.gov/srmors/certificates/1649B.pdf. Antibodies against phospho-AMPK and AMPK were purchased from Cell Signaling Technology (Danvers, MA, USA). Antibodies against C1QTNF6 and GAPDH were purchased from Abcam (Cambridge, MA, USA) and Beyotime (Shanghai, China), respectively. Reagents for western blot analysis and immunohistological staining were purchased from Beyotime. All primers used in this study were synthesized by Synbio Technologies (Suzhou, China).

### Animal treatment

Male C57BL/6 mice (6–8 weeks, 20–22 g) were purchased from SLAC Laboratory Animals (Shanghai, China). All mice were fed in a specific pathogen-free facility with normal chow and water and with a 12-h light-dark cycle. All protocols for the animal experiments were ethically reviewed and approved by the Animal Care and Use Committee of Zhongshan Hospital, Fudan University. The PM was suspended in sterile PBS to a final concentration of 4 mg/cm^3^. Mice were intratracheally administered with 100 μg PM in 25 μL sterile PBS per day for 2 consecutive days to establish the acute PM-exposed mouse model, as we previously reported [5, 8]. A chemically modified antagomir complementary to miR-29b-3p designed to inhibit miR-29b-3p expression, and the negative control antagomirs, were purchased from GenePharma (Shanghai, China). The antagomirs (150 μg in 0.2 mL sterile PBS per mouse) were delivered through the tail vein of mice 24 h prior to the first PM exposure.

### Cell culture and treatment

HBECs were obtained from the Chinese Academy of Sciences (Shanghai, China). Murine lung type II epithelial cells (MLE-12) and murine macrophages (RAW264.7) were obtained from American Type Culture Collection (Manassas, VA, USA). The cells were cultured in RPMI-1640 medium (Hyclone, Logan, UT, USA) supplemented with 10% fetal bovine serum (Gibco, Waltham, MA, USA) and 50 U/mL penicillin and streptomycin (Gibco) at 37°C in a culture chamber containing 5% CO_2_. The PM was suspended in sterile PBS at a final concentration of 10 mg/cm^3^. The three cell types were treated with PM at 300 μg/cm^3^ for 24 h. Furthermore, HBECs were treated with different doses of PM (50, 100, 200, and 300 μg/cm^3^) for 24 h and 300 μg/cm^3^ PM for different durations (3, 6, 12, and 24 h).

### Western blot analysis

Cells or lung tissues from different groups were lysed in radioimmunoprecipitation assay buffer (RIPA) buffer containing 1% phenylmethanesulfonyl fluoride (PMSF) and 1% phosphatase inhibitors (Biotool, Houston, TX, USA). The concentration of proteins was determined using BCA Protein Assay kit. Protein (30 μg) from each group was subjected to electrophoresis on 10% sodium dodecyl sulphatepolyacrylamide gels, and then transferred to polyvinylidene difluoride (PVDF) membranes. After blocking with western blocking buffer for 1 h at room temperature, each membrane was incubated with antibody against phospho-AMPK, AMPK, C1QTNF6, or GAPDH (1:1000 dilution) at 4°C overnight. The membrane was then washed with TBST and incubated with anti-rabbit or anti-mouse horseradish peroxidase (HRP)-conjugated secondary antibody (1:5000 dilution) for 1 h at room temperature. Thereafter, the results were visualized using ECL reagents (Thermo Scientific, Waltham, MA, USA) with a Bio-Rad Laboratories system (Hercules, CA, USA). The optical densities of the protein bands were quantified using AlphaEaseFC v4.0 software.

### Measurement of miRNA and mRNA expression

Total RNA was extracted using Trizol reagent (Invitrogen, Grand Island, NY, USA) according to the manufacturer's instructions, and was quantified by a NanoVue Plus spectrophotometer (GE Healthcare, Chicago, IN, USA). MiRNAs were reverse transcribed using a miRNA First Strand cDNA Synthesis Kit (Tailing Reaction, Sangon Biotech, Shanghai, China), while mRNAs were reverse transcribed into cDNA using Reverse Transcription Reagents (TaKaRa Bio, Shiga, Japan), according to the manufacturer’s protocols. Quantitative real-time (RT)-PCR was conducted using TB Green^TM^ Premix Ex Taq^TM^ (TaKaRa Bio) on a RT-PCR system (Bio-Rad Laboratories). U6 and GAPDH were used as internal controls to determine the relative expression of miRNAs and mRNAs, respectively. The relative expression was reported as a 2^−△△Ct^ value. To construct a miRNA panel, 92 potential miRNAs most likely to be relevant to inflammation were selected based on the miRNA list provided by Qiagen (Valencia, CA, USA). The primer sequences for all miRNAs and mRNAs are shown in Supplementary Table 1.

### Enzyme-linked immunosorbent assay (ELISA)

The concentrations of IL-1β, IL-6, and IL-8 in the BALF were measured using ELISA kits (4A Biotech, Beijing, China). The experimental procedures were performed according to the manufacturer’s protocols [5].

### Transfection with siRNA, miRNA mimics, and miRNA inhibitors

Three custom-designed AMPK siRNAs, three custom-designed C1QTNF6 siRNAs, a miR-29b-3p mimic, a miR-29b-3p inhibitor, and their negative controls were obtained from GenePharma (Shanghai, China). The HBECs were transfected with siRNA or miRNA using Lipofectamine 2000 (Invitrogen), according to the manufacturer’s protocol. The inhibition efficiencies of the siRNA were measured by RT-PCR and the AMPK and C1QTNF6 siRNAs with the best inhibition efficiencies were used for the subsequent experiments. After 24 h of transfection with siRNA or miRNA, HBECs were stimulated with PM to assess the inflammatory responses.

### Overexpression vector construction and transfection

The full length of the C1QTNF6 gene was amplified from genomic DNA and cloned into a PHY-023 vector (Clonetech, TaKaRa Bio, Shiga, Japan). All procedures to construct and identify the plasmid were performed by Hanyin Biotechnology Limited Company (Shanghai, China). Thereafter, C1QTNF6-overexpressing or negative control plasmid along with pMD2G and psPAX2 were used to transfect HEK293T cells for lentivirus packaging. The lentiviruses were purified and used to transfect HBECs. At 48 h after transfection, the cells with stably integrated constructs were selected with 1 μg/mL puromycin for 7 days. The overexpression efficiency was confirmed using RT-PCR and western blot analysis.

### RNA sequencing and bioinformatics analysis

HBECs were transfected with 29b-m or negative control mimic for 24 h and then stimulated with or without PM for another 24 h. The total RNA was isolated from the four groups (29b-m, NC-m, 29b-m + PM, and NC-m + PM groups) using Trizol reagent according to the manufacturer's instructions. The concentration and integrity of RNA was evaluated using an Agilent 2100 Bioanalyzer platform (Agilent Technologies, CA, USA). Thereafter, mRNA was enriched using poly-T oligo attached magnetic beads and reverse transcribed using random primers. Next, the double-stranded cDNA was ligated with an adaptor and amplified to create a cDNA library. The RNA sequencing was conducted using a BGISEQ-500 platform and the high-quality reads were aligned to the human reference genome (GRCh38). The differentially expressed genes from the NC-m *vs.* 29b-m groups, NC-m + PM *vs.* 29b-m + PM groups, and NC-m *vs.* NC-m + PM groups, were defined based on log2 fold change ≥ 1 and adjusted p-value ≤ 0.05, and were further analyzed to identify the target genes of miR-29b-3p in HBECs combining with TargetScan database (http://www.targetscan.org/vert_72/).

### Histological analysis

Lungs from different mouse groups were fixed in 4% paraformaldehyde overnight, and they were then embedded in paraffin and sliced into 4 μm thick sections using a routine histologic procedure. The lung tissue sections were subsequently stained with hematoxylin and eosin (H&E) according to the manufacturer’s instructions. Pathologic changes were imaged as reported previously [39]. The BALFs were centrifuged to collect the supernatants for ELISAs. Cell pellets were resuspended in 0.5 mL PBS and the total cell number was counted. Thereafter, 100 μL resuspended cells were spun to a slide, fixed with 4% paraformaldehyde overnight, and stained with H&E. The numbers of inflammatory cells were counted, as we previously reported [5].

### Immunohistological analysis

Lungs from different mouse groups were fixed, embedded, and sliced for immunohistochemical staining. After dewaxing and blocking, the lung tissue sections were incubated with primary antibody against C1QTNF6 (1:100 dilution) overnight at 4°C. The next day, after washing with PBS, the sections were incubated with HRP-conjugated secondary antibody and then visualized using 3, 3′-diaminobenzidine (DAB). Images were obtained using a light microscope (Olympus, Tokyo, Japan).

### Dual luciferase reporter assay

Two conserved miR-29b-3p binding sites in the 3'-UTR of C1QTNF6 were predicted using the TargetScan database. The 3'-UTR of C1QTNF6 was amplified from genomic DNA and inserted into a pmiGLO vector (Promega, Madison, WI, USA) to construct a C1QTNF6-3'-UTR-wild type (WT) plasmid. Each of the two predicted miR-29b-3p binding sites in the 3'-UTR of C1QTNF6 was mutated separately and cloned into the pmiGLO vector to form a C1QTNF6-3'-UTR-mutA and C1QTNF6-3'-UTR-mutB plasmid, respectively. Furthermore, both binding sites were mutated and cloned into the pmiGLO vector to form a C1QTNF6-3'-UTR-mutAB plasmid. The procedures to construct and identify these plasmids were performed by Hanyin Biotechnology Limited Company (Shanghai, China). Each plasmid along with 29b-m or NC-m were transfected into HBECs cells using Lipofectamine 2000, according to the manufacturer’s protocol. Luciferase activity was measured 24 h later using a Dual-Luciferase® reporter assay system (Promega) with luciferase activity being normalized to Renilla. Normalized luciferase activity was expressed as a percentage of the negative control for the same constructs.

### Statistical analysis

The data are presented as mean ± SEM and analyzed using SPSS v19.0 software (IBM Corp., Armonk, NY, USA). Data of two groups were compared using the unpaired Student’s t-test, while data of three or more groups were compared using one-way analysis of variance (ANOVA). P value ≤ 0.05 was considered significant.

## Supplementary Material

Supplementary Figures

Supplementary Table 1
