# Supplementary material for: MiR-29b-3p promotes particulate matter-induced inflammatory responses by regulating the C1QTNF6/AMPK pathway
Source: Aging (Albany NY). 2020 Jan 18;12(2):1141–58. doi: 10.18632/aging.102672 (PMC7053628; doi:10.18632/aging.102672)
Supplement: Supplementary Figures [file aging-12-102672-s002..pdf]

SUPPLEMENTARY FIGURE

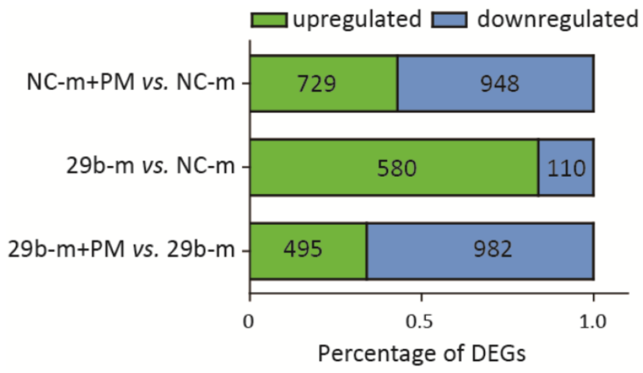

Supplementary Figure 1. The number of differentially expressed genes was identified in NC-m *vs.* 29b-m group, NC-m + PM *vs.* 29b-m + PM group, and NC-m *vs.* NC-m + PM group.

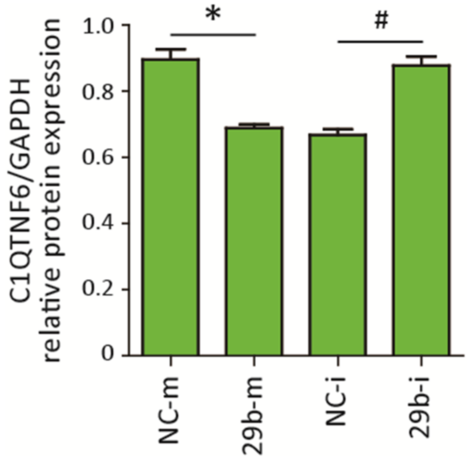

Supplementary Figure 2. The optical densities of protein bands for Figure 4F. Values were shown as mean ± SEM; *, P<0.05, compared with the NC-m group; #, P<0.05, compared with the NC-i group; n=3.

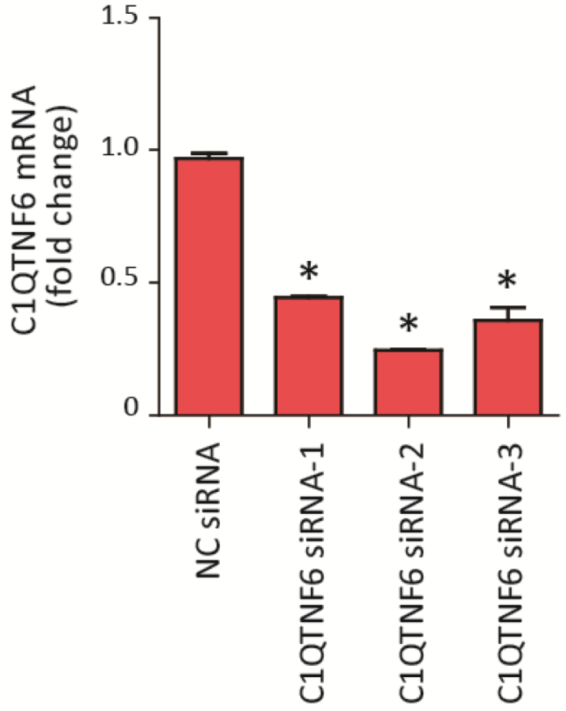

Supplementary Figure 3. The optimum C1QTNF6 siRNA was identified according to the inhibition efficiency of three designed siRNAs using RT-PCR. The C1QTNF6 siRNAs were transfected into HBECs and the optimum C1QTNF6 siRNA was selected using real-time PCR. Values were shown as mean ± SEM; *, P<0.05, compared with the NC siRNA group; n=3.
