# Supplementary material for: MiR-29b-3p promotes particulate matter-induced inflammatory responses by regulating the C1QTNF6/AMPK pathway
Source: Aging (Albany NY). 2020 Jan 18;12(2):1141–58. doi: 10.18632/aging.102672 (PMC7053628; doi:10.18632/aging.102672)
Supplement: Supplementary Table 1 [file aging-12-102672-s001..docx]

**Supplementary Table1. Primers of miRNAs and mRNAs**

| **MiRNAs/mRNAs** | **Primer sequences** |
| --- | --- |
| let-7a-5p-F  let-7b-5p-F  let-7c-3p-F  let-7c-5p-F  let-7d-5p-F  let-7e-5p-F  let-7f-5p-F  let-7g-5p-F  let-7i-5p-F  miR-101-3 p-F  miR-125a-5p-F  miR-125b-5p-F  miR-130a-3p-F  miR-130b-3p-F  miR-1324-F  miR-144-3p-F  miR-145-5p-F  miR-15a-5p-F  miR-15b-5p-F  miR-16-5p-F  miR-17-5p-F  miR-181a-5p-F  miR-181b-5p-F  miR-181c-5p-F  miR-181d-3p-F  miR-181d-5p-F  miR-186-5p-F  miR-195-5p-F  miR-19a-3p-F  miR-19b-3p-F  miR-202-3p-F  miR-20a-5p-F  miR-20b-5p-F  miR-211-5p-F  miR-21-5p-F  miR-23a-3p-F  miR-23b-3p-F  miR-29a-3p-F  miR-29b-3p-F  miR-29c-3p-F  miR-300-F  miR-301a-3p-F  miR-301b-3p-F  miR-301b-5p-F  miR-302a-3p-F  miR-302b-3p-F  miR-302c-3p-F  miR-30a-5p-F  miR-30b-5p-F  miR-30c-5p-F  miR-30d-5p-F  miR-30e-5p-F  miR-340-5p-F  miR-34a-5p-F  miR-34c-5p-F  miR-372-F  miR-373-3p-F  miR-374a-5p-F  miR-381-3p-F  miR-381-5p-F  miR-410-3p-F  miR-410-5p-F  miR-424-5p-F  miR-449a-F  miR-449b-5p-F  miR-454-3p-F  miR-497-5p-F  miR-511-F  miR-513b-F  miR-519c-3p-F  miR-519d-3p-F  miR-519d-5p-F  miR-520d-3p-F  miR-520e-3p-F  miR-520e-5p-F  miR-524-5p-F  miR-543-F  miR-545-3p-F  miR-548c-3p-F  miR-548d-3p-F  miR-548e-3p-F  miR-548e-5p-F  miR-590-5p-F  miR-607-F  miR-655-F  miR-656-3p-F  miR-656-5p-F  miR-875-3p-F  miR-93-5p-F  miR-9-5p-F  miR-98-3p-F  miR-98-5p-F  IL-1β-F  IL-1β-R  IL-6-F  IL-6-R  IL-8-F  IL-8-R  C1QTNF6-F  C1QTNF6-R  AMPK-F  AMPK-R  GAPDH-F  GAPDH-R | 5'-CGCGCTGAGGTAGTAGGTTGTATAGTT-3'  5'-CGCTGAGGTAGTAGGTTGTGTGGTT-3'  5'-CGCCTGTACAACCTTCTAGCTTTCC-3'  5'-GCGCTGAGGTAGTAGGTTGTATGGTT-3'  5'-CGCGAGAGGTAGTAGGTTGCATAGTT-3'  5'-CGCGTGAGGTAGGAGGTTGTATAGTT-3'  5'-CGGCGCTGAGGTAGTAGATTGTATAGTT-3'  5'-AGCGCTGAGGTAGTAGTTTGTACAGTT-3'  5'-CGCGTGAGGTAGTAGTTTGTGCTGTT-3'  5'-CGCGCGTACAGTACTGTGATAACTGAA-3'  5'-TCCCTGAGACCCTTTAACCTGTGA-3'  5'-GCTCCCTGAGACCCTAACTTGTGA-3'  5'-CGCCAGTGCAATGTTAAAAGGGCAT-3'  5'-CGCAGTGCAATGATGAAAGGGCAT-3'  5'-CCGCCAGACAGAATTCTATGCACTTTC-3'  5'-CCGCGCGTACAGTATAGATGATGTACT-3'  5'-GTCCAGTTTTCCCAGGAATCCCT-3'  5'-CCGCTAGCAGCACATAATGGTTTGTG-3'  5'-CGCTAGCAGCACATCATGGTTTACA-3'  5'-CGCTAGCAGCACGTAAATATTGGCG-3'  5'-GCCAAAGTGCTTACAGTGCAGGTAG-3'  5'-AACATTCAACGCTGTCGGTGAGT-3'  5'-AACATTCATTGCTGTCGGTGGGT-3'  5'-CGAACATTCAACCTGTCGGTGAGT-3'  5'-CCACCGGGGGATGAATGTCA-3'  5'-CGAACATTCATTGTTGTCGGTGGGT-3'  5'-CCGCAAAGAATTCTCCTTTTGGGCT-3'  5'-ACGCTAGCAGCACAGAAATATTGGC-3'  5'-CGCGTGTGCAAATCTATGCAAAACTGA-3'  5'-ACGTGTGCAAATCCATGCAAAACTGA-3'  5'-ACGAGAGGTATAGGGCATGGGAA-3'  5'-CGCGTAAAGTGCTTATAGTGCAGGTAG-3'  5'-CGCAAAGTGCTCATAGTGCAGGTAG-3'  5'-CTTCCCTTTGTCATCCTTCGCCT-3'  5'-CCGCGTAGCTTATCAGACTGATGTTGA-3'  5'-CCGATCACATTGCCAGGGATTTCC-3'  5'-CCGATCACATTGCCAGGGATTACC-3'  5'-GCGTAGCACCATCTGAAATCGGTTA-3'  5'-GCGCTAGCACCATTTGAAATCAGTGTT-3'  5'-CGCGTAGCACCATTTGAAATCGGTTA-3'  5'-CGCTATACAAGGGCAGACTCTCTCT-3'  5'-CGCGCAGTGCAATAGTATTGTCAAAGC-3'  5'-CGCCAGTGCAATGATATTGTCAAAGC-3'  5'-GCTCTGACGAGGTTGCACTACT-3'  5'-CGCTAAGTGCTTCCATGTTTTGGTGA-3'  5'-CCGCGTAAGTGCTTCCATGTTTTAGTAG-3'  5'-CCGTAAGTGCTTCCATGTTTCAGTGG-3'  5'-CGCTGTAAACATCCTCGACTGGAAG-3'  5'-ACGCTGTAAACATCCTACACTCAGCT-3'  5'-CGCTGTAAACATCCTACACTCTCAGC-3'  5'-CGTGTAAACATCCCCGACTGGAAG-3'  5'-CGCGTGTAAACATCCTTGACTGGAAG-3'  5'-CGCGCGTTATAAAGCAATGAGACTGATT-3'  5'-CTGGCAGTGTCTTAGCTGGTTGT-3'  5'-CGAGGCAGTGTAGTTAGCTGATTGC-3'  5'-AAAGTGCTGCGACATTTGAGCGT-3'  5'-CGAAGTGCTTCGATTTTGGGGTGT-3'  5'-GCGCGCGTTATAATACAACCTGATAAGTG-3'  5'-CGCTATACAAGGGCAAGCTCTCTGT-3'  5'-CGAGCGAGGTTGCCCTTTGTATAT-3'  5'-CGCGAATATAACACAGATGGCCTGT-3'  5'-ACGAGGTTGTCTGTGATGAGTTCG-3'  5'-ACCGCAGCAGCAATTCATGTTTTGAA-3'  5'-CGTGGCAGTGTATTGTTAGCTGGT-3'  5'-CCAGGCAGTGTATTGTTAGCTGGC-3'  5'-CCGCGTAGTGCAATATTGCTTATAGGGT-3'  5'-ACAGCAGCACACTGTGGTTTGT-3'  5'-CGGTGTCTTTTGCTCTGCAGTCA-3'  5'-CGCGTTCACAAGGAGGTGTCATTTAT-3'  5'-GCGCGAAAGTGCATCTTTTTAGAGGAT-3'  5'-CGCAAAGTGCCTCCCTTTAGAGTG-3'  5'-CCTCCAAAGGGAAGCGCTTTCT-3'  5'-CGAAAGTGCTTCTCTTTGGTGGGT-3'  5'-GCGAAAGTGCTTCCTTTTTGAGGG-3'  5'-GCGCTCAAGATGGAAGCAGTTTCTG-3'  5'-CCGCTACAAAGGGAAGCACTTTCTC-3'  5'-CGAAACATTCGCGGTGCACTTCTT-3'  5'-GCGCTCAGCAAACATTTATTGTGTGC-3'  5'-CGCCGCGCAAAAATCTCAATTACTTTTGC-3'  5'-CGCGCAAAAACCACAGTTTCTTTTGC-3'  5'-GCGCGAAAAACTGAGACTACTTTTGCA-3'  5'-GCAAAAGCAATCGCGGTTTTTGC-3'  5'-ACGCGGAGCTTATTCATAAAAGTGCAG-3'  5'-CGCGCGGTTCAAATCCAGATCTATAAC-3'  5'-CCGCGCGATAATACATGGTTAACCTCTTT-3'  5'-GCGCGCGAATATTATACAGTCAACCTCT-3'  5'-AGGTTGCCTGTGAGGTGTTCA-3'  5'-CGCCTGGAAACACTGAGGTTGTG-3'  5'-GCAAAGTGCTGTTCGTGCAGGTAG-3'  5'-CCGCGTCTTTGGTTATCTAGCTGTATGA-3'  5'-GCGCGCTATACAACTTACTACTTTCCC-3'  5'-CGCGCGTGAGGTAGTAAGTTGTATTGTT-3'  5'-TGGCAATGAGGATGACTTGT-3'  5'-TGGTGGTCGGAGATTCGTA-3'  5'-TTCGGTCCAGTTGCCTTCT-3'  5'-GGTGAGTGGCTGTCTGTGTG-3'  5'-TTGCCAAGGAGTGCTAAAGAA-3'  5'-GCCCTCTTCAAAAACTTCTCC-3'  5'-TGATGCTGGACCTGGCCTACG-3'  5'-AAGTCGTTGCTGTAGATGGCGTTC-3'  5'-TTGTATGCAGGCCCAGAGGT-3'  5'-TGGGATCCACCTGCAGCATA-3'  5'-CCACCCATGGCAAATTCCATGGCA-3'  5'-TCTACACGGCAGGTCAGGTCCACC-3' |
